# Current scenario of machine learning applications to hydrothermal liquefaction via bibliometric analysis

**DOI:** 10.12688/f1000research.156514.1

**Published:** 2024-10-04

**Authors:** Tossapon Katongtung, Somboon Sukpancharoen, Sakprayut Sinthupinyo, Nakorn Tippayawong

**Affiliations:** 1 Graduate PhD Program in Energy Engineering, Faculty of Engineering, Chiang Mai University, Chiang Mai, Thailand; 2 Department of Mechanical Engineering, Faculty of Engineering, Chiang Mai University, Chiang Mai, Thailand; 3 Department of Agricultural Engineering, Faculty of Engineering, Khon Kaen University, Khon Kaen, 40002, Thailand; 4 Siam Research and Innovation Co., Ltd, Bangkok, Thailand

**Keywords:** AI, Clean energy, Data analytics, Climate action, bibliometric analysis

## Abstract

**Background:**

Energy shortages and global warming have been significant issues throughout history. Therefore, the search for environmentally friendly renewable energy sources is crucial for achieving sustainability. Biomass energy is gaining global attention as a renewable energy option, particularly through the process of hydrothermal liquefaction, which converts biomass into bio-crude oil.

**Methods:**

Hydrothermal liquefaction is a complex process that is challenging to explain, leading to research on machine learning models for this process. These models aim to predict values and investigate the impact of variables on the hydrothermal liquefaction process. However, the development of machine learning in hydrothermal liquefaction is still limited due to its novelty and the time required for comprehensive study. Thus, the objective of this study was to analyze relevant publications in the Scopus database, focusing on indexed ML and HTL keywords, to understand keyword associations and co-citations.

**Results:**

The results reveal an increasing trend in the study of ML in the HTL process, with a growing interest from various countries.

**Conclusion:**

Notably, China currently holds the largest share of ML research in HTL processes, with most published works falling within the field of engineering. The keyword “liquefaction” emerges as the most popular term in these publications.

## Introduction

Currently, numerous countries worldwide are actively seeking alternative energy sources to replace depleted resources and ensure environmental preservation amidst the era of global warming. ^1^^–^^3^ Biomass stands as a prominent alternative in the realm of renewable energy, referred to as biomass energy. ^4^ Biomass energy refers to the energy derived from various organic materials that serve as natural energy sources. These materials encompass a wide range of substances, including organic waste, agricultural residues, industrial byproducts, manure, and fuel crops such as rice husks, rice straw, bagasse, sugar cane leaves and shoots, wood, wood chips, fibers, palm kernel shells, cassava residue, corn cobs, husks, coconut shells, and more. The conversion of biomass into usable energy involves processes such as fermentation, combustion, hydrothermal treatment, or other methodologies, which transform biomass into heat or gas for energy utilization. ^5^^,^^6^ Additionally, there exists a biomass processing method known as hydrothermal liquefaction, which converts wet biomass into bio-crude oil. This process involves subjecting the biomass to high-temperature and high-pressure conditions in a liquid water environment, resulting in the production of bio-crude oil. ^7^^,^^8^

Hydrothermal liquefaction (HTL) is a thermal depolymerization process that facilitates the conversion of wet biomass and other macromolecules into crude oil, commonly known as bio-crude oil. This transformation occurs under medium temperature and high-pressure conditions, enabling the efficient conversion of the biomass feedstock. ^9^^,^^10^ Bio-crude oil exhibits a high energy density, characterized by a higher heating value ranging from 33.8 to 36.9 MJ/kg. It typically contains 5-20 wt% oxygen, along with renewable chemicals. This composition makes bio-crude oil an attractive renewable energy source with significant potential for various applications. ^11^ The study of various variables and their effects under HTL processing conditions is challenging and intricate. Consequently, researchers worldwide have shown keen interest in investigating the behavior of variables within the HTL environment. Machine learning has emerged as the most popular technology to address such difficult and complex problems efficiently. It offers promising avenues for understanding and optimizing HTL processes through data-driven analysis and predictive modeling.

Machine Learning (ML) can be considered as the learning component of a machine. It serves as the foundational element of Artificial Intelligence (AI), acting as the “brain” that enables AI systems to acquire knowledge and exhibit intelligent behavior. AI utilizes ML techniques to develop and enhance its intelligent capabilities, allowing it to learn from data, recognize patterns, make predictions, and adapt to new information or situations. In essence, ML forms an integral part of the AI framework, enabling the creation and manifestation of intelligence in AI systems. ^12^^,^^13^ Indeed, ML is commonly associated with the learning models of artificial intelligence. Unlike traditional programming, where humans explicitly define rules and instructions, ML involves programming AI systems to learn from data. Once programmed, the machine leverages the available data to train and refine its own intelligence through practice and iterative processes. It gradually improves its performance and ability to make informed decisions without explicit human intervention. ML has found extensive applications across various domains, including medicine, education, economy, and engineering. In medicine, it is used for diagnostics, personalized treatments, and drug discovery. In education, it aids in adaptive learning and intelligent tutoring systems. In the economy, it assists with data analysis, forecasting, and fraud detection. In engineering, it facilitates automation, optimization, and predictive maintenance. The widespread adoption of ML in both research and everyday life has opened up new possibilities and opportunities for leveraging data-driven intelligence in solving complex problems and making informed decisions. ^13^^–^^18^

Moreover, ML is extensively applied in the field of energy to predict and establish correlations among variables in highly complex processes that cannot be easily explained through conventional means. For instance, a study by Onsree and Tippayawong (2020) demonstrated the development of a ML application for predicting yields of solid products obtained from biomass torrefaction. This model achieved a remarkable accuracy with an R2 value of approximately 0.9. Furthermore, the application also shed light on the interrelationships between variables within the torrefaction process, providing valuable insights into the underlying mechanisms. Such applications of ML in energy-related research not only enhance predictive capabilities but also contribute to a deeper understanding of complex processes and their underlying dynamics. ^19^ Additionally, Phromphithak et al. (2021) conducted a study where they employed ML techniques to predict the production of cellulose-rich materials during biomass pretreatment using ionic liquid solvents. The developed model exhibited a high accuracy, with an R2 value of 0.94. Moreover, the study successfully elucidated the behavior of ionic liquids and their impact on the ML -based pretreatment process. Ionic liquid (IL) pretreatment is an effective method for processing lignocellulosic biomass, enhancing its conversion into biofuels and chemicals. ILs, which are salts in liquid form at low temperatures, can disrupt the complex structure of biomass by dissolving lignin and reducing the crystallinity of cellulose. This makes cellulose more accessible for enzymatic hydrolysis or catalytic processes. ILs offer the advantage of selective dissolution, allowing for better isolation of cellulose and hemicellulose. Furthermore, ILs can be recycled, reducing waste and costs, making them a promising option for sustainable biomass pretreatment. This research demonstrates the efficacy of ML in providing accurate predictions and uncovering the intricate relationships between variables, leading to a better understanding of the effects of ionic liquids in biomass. ^20^ In a study by Prasertpong et al. (2022), the researchers investigated the synergistic effects observed during the co-pyrolysis of biomass and plastic waste using ML techniques. The aim of the study was to gain insights into the variables that influence the co-pyrolysis process. By employing machine learning, the researchers were able to uncover and comprehend the intricate relationships and interactions between these variables, shedding light on the synergistic effects observed during the co-pyrolysis process. This study highlights the potential of ML in providing valuable insights and understanding complex processes involving biomass and plastic waste co-pyrolysis. ^21^ Furthermore, there have been studies that utilize ML in the context of HTL processes. In a previous research endeavor, an ML model was developed for predicting biocrude yields and higher heating values obtained from HTL of wet biomass and waste materials. The model achieved a high precision with an R2 value of nearly 0.9. Additionally, it elucidated the relationships among variables that directly and indirectly impact the HTL process. This study showcases the potential of ML in accurately predicting and comprehending the complex interplay of variables in HTL processes, contributing to a deeper understanding of the factors influencing HTL and enabling more efficient and effective utilization of wet biomass and waste for biocrude production. ^22^ There are other studies related to ML in HTL processes a number of similar. ^23^^,^^24^ Indeed, the development of ML in the field of HTL is relatively new and holds great potential for further study and exploration in the future. Consequently, the collection and comprehensive search for research pertaining to the development of ML in the HTL process become crucial and should be given significant emphasis. This endeavor would facilitate the identification of existing studies, trends, and advancements in the application of ML techniques specifically for HTL, thereby laying the groundwork for future research and advancements in this domain. By focusing on this area of research, researchers can deepen their understanding of the potential of ML in HTL and further contribute to its development and utilization in the pursuit of sustainable and efficient energy production.

Bibliometric analysis involves a systematic statistical examination of research data extracted from extensive databases, aiming to evaluate various aspects of scholarly work. This analysis serves as a valuable tool for measuring research quality, including the productivity and impact of individual researchers and institutions. By employing bibliometric methods, researchers can quantitatively assess factors such as publication output, citation counts, collaboration networks, journal rankings, and other bibliographic indicators. These analyses provide valuable insights into the performance and influence of researchers and institutions within specific fields of study, contributing to the evaluation and comparison of research outcomes across various domains. ^25^ Bibliometric analysis is a research methodology that emphasizes quantitative and statistical investigations. It enables the description and analysis of various aspects of scholarly publications or literature, such as the type of publication, the countries, institutions, and authors associated with the research. By conducting long-term studies, bibliometric analysis allows researchers to identify trends in the growth or decline of research within specific fields. Consequently, bibliometric data serves as a valuable foundation for monitoring scientific and technological research, providing insights into the progress and development of knowledge in various domains. ^26^ Bibliographic analysis typically involves selecting a set of keywords to search relevant literature databases. These keywords are chosen based on the topic and title of the articles to retrieve information related to the conceptual framework and writing style within a specific field of study. Electronic bibliographic databases offer vast opportunities for efficient and rapid access to a wide range of scholarly resources. By utilizing these databases, researchers can benefit from an extensive collection of literature and gain quicker access to relevant information for their studies. This enables more efficient literature review and analysis, aiding in the advancement of research in a particular area. ^27^

The objective of this study was to perform a bibliographic analysis using publications related to ML and HTL keywords indexed in the Scopus database. The analysis employed a quantitative methodology to examine the bibliography of published articles in the field. To accomplish this objective, the study utilized VOSviewer version 1.6.20, a bibliometric analysis tool, for conducting the bibliographic analysis. VOSviewer enables visualizations and quantitative analyses of bibliographic data, facilitating the exploration of relationships, trends, and patterns within the literature. ^28^^,^^29^ Based on the available literature review, this study represents the first bibliographic analysis conducted to evaluate research trends specifically in the development of ML within the context of HTL. As the research on HTL-based ML development is still in its early stages and relatively limited at present, this study fills an important knowledge gap in the field. The outcomes of this analysis will contribute significantly to the formulation of a comprehensive research plan by identifying potential future research directions and fostering collaborative relationships among researchers in this emerging field.

## Methods

Bibliometrics is a research methodology that involves the analysis of quantitative and statistical data. It is commonly used to examine various relationships, such as the connections between authors, the relationship between a research subject and an author’s work, scholarly works themselves, scholarly citations, and citation tracking. By employing bibliometric techniques, researchers can gain insights into the patterns and dynamics of scholarly communication, collaboration, and impact within a particular field or discipline. It provides a systematic approach to understanding the scholarly landscape and can be utilized to assess research productivity, influence, and trends. ^30^ Bibliometric analysis offers a distinct perspective that complements more comprehensive analyses. It enables researchers to categorize and analyze extensive amounts of data derived from research conducted over a specific timeframe. By employing quantitative techniques, bibliometric analysis can help mitigate or minimize researcher bias, unlike systematic reviews that often rely on qualitative methods. The objectivity of bibliometric analysis reduces the potential for interpretation bias introduced by scholars from diverse academic backgrounds. This allows for a more objective and standardized assessment of research output, impact, and trends, providing valuable insights into the scholarly landscape. ^28^^,^^31^

**Figure 1.**
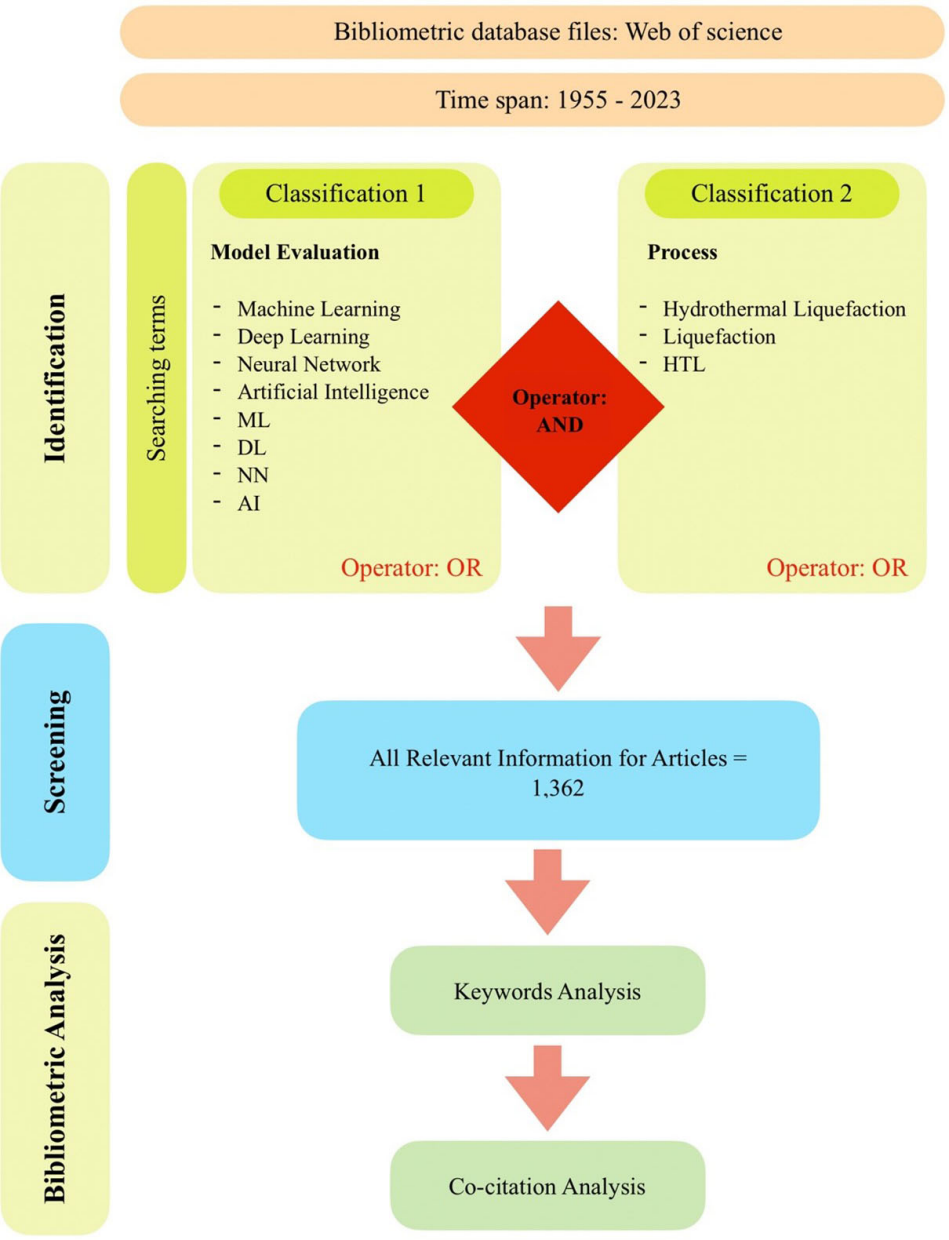
Flow chart of the search approach.

In this study, bibliographic analysis was employed to investigate and validate the prevailing trends in the research on ML development within HTL processes. The Scopus database was selected for this analysis due to its widespread usage, reliability, and comprehensive coverage across various disciplines. Scopus is known for its extensive collection of scholarly literature, making it a suitable choice for conducting bibliometric studies across diverse research areas. By utilizing the Scopus database, the study could effectively assess and examine the current state of ML development in HTL processes and gain valuable insights into the research landscape in this domain. ^32^^,^^33^ Indeed, the databases available in Scopus offer access to a vast number of documents, providing a wealth of information for bibliometric analysis. Scopus is renowned for its comprehensive coverage of scholarly literature, encompassing a wide range of disciplines and research fields. This extensive coverage ensures that researchers can access a substantial volume of documents relevant to their study. Furthermore, Scopus provides robust citation information, including citation counts and citation networks, which can be crucial for analyzing the impact and influence of research articles. The availability of comprehensive document and citation data in Scopus enhance the accuracy and reliability of bibliometric analyses and contribute to a more comprehensive understanding of research trends and dynamics. ^34^ Scopus is a comprehensive database that provides researchers with a range of resources for exploring and evaluating scholarly publications, patents, clinical trials, and policy documents. In this study, the Scopus database was utilized to conduct the bibliographic analysis. Keywords were employed to search for relevant publications based on their publication name, abstract, and author-provided keywords. The search criteria encompassed a set of specific keywords related to “Machine Learning,” “Deep Learning,” “Neural Network,” “Artificial Intelligence,” “ML,” “DL,” “NN,” and “AI,” as well as keywords related to “Hydrothermal Liquefaction,” “Liquefaction,” and “HTL.” The subject areas covered in this study include energy, biomass, engineering, computer science, chemistry, biology, and the environment. The search keywords were set to include publications from the year 1955 up to June 2023, with data downloaded on June 21, 2023. The inclusion criteria for this study encompassed original articles published in the English language. These specific parameters and criteria were employed to ensure a focused and comprehensive analysis of the relevant literature in the field of ML development in HTL processes. Figure 1 shows the search technique used in this study to identify appropriate articles from the Scopus database. The complete bibliography data was downloaded in.csv format from the Scopus database. VOSviewer was used in bibliometric analysis in this work.

## Results

### Main data and annual publication growth

**Figure 2.**
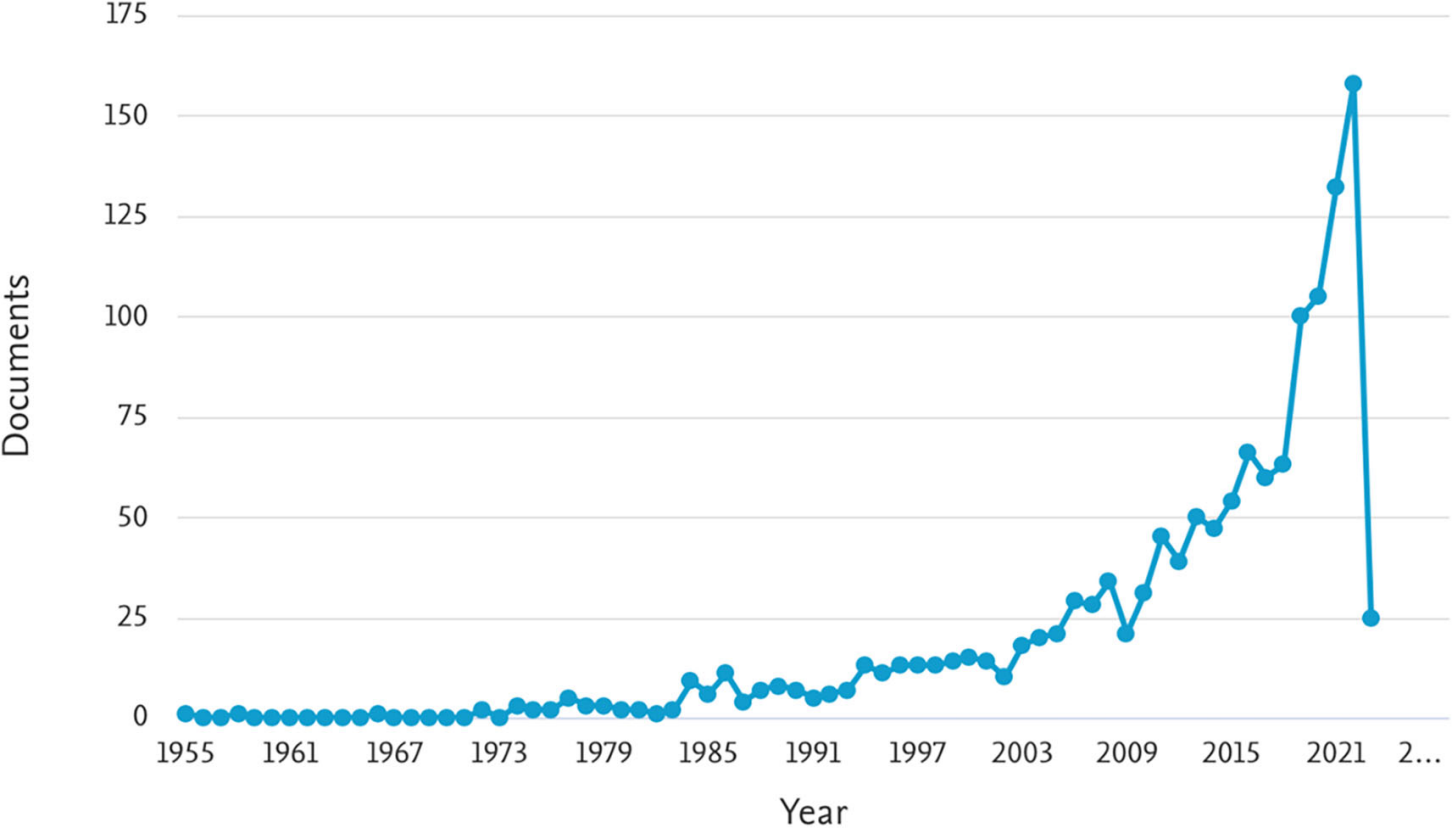
Document by year. (via Scopus on July 1, 2023).

Table 1 provides an overview of the number of documents related to ML in the HTL process, categorized by year of publication from 1955 to 2023. The table shows the number of documents found for each year, indicating the volume of research conducted in this area. Figure 2 illustrates the trend in the number of documents related to ML in the HTL process per year. The graph indicates a consistent increase in the number of publications since 2003, with a significant surge observed from 2018 onwards. Notably, in 2018, 2019, 2020, 2021, and 2022, the number of documents were 63, 100, 105, 132, and 158, respectively. This trend suggests a growing interest and popularity in the study of ML in the HTL process, particularly in recent years. The findings from Figure 2 indicate that ML in the HTL process has garnered substantial attention and continues to gain momentum, highlighting its significance and potential for further advancements in research and applications.

**Table 1.** Main information.

Description	Results
**Information about data**	
Time span	1955-2023
Documents	1,362
Citation	17,068
H-index	69
References	33,076
**Document type**	
Article	1,148
Conference Paper	155
Conference Review	20
Book Chapter	17
Review	17
Book	3
Data Paper	2

Statistical data on the number of documents related to ML in the HTL process.

### Top 10 in various fields

**Figure 3.**
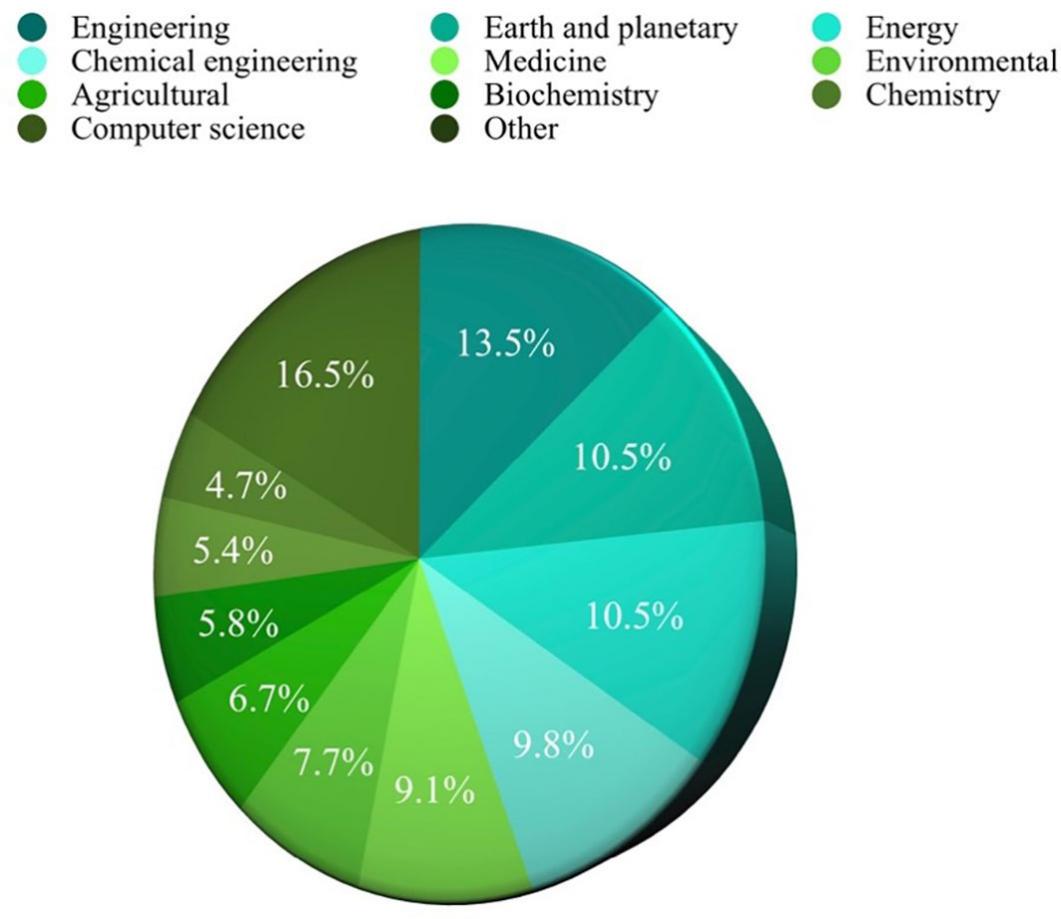
Published subject area.

**Figure 4.**
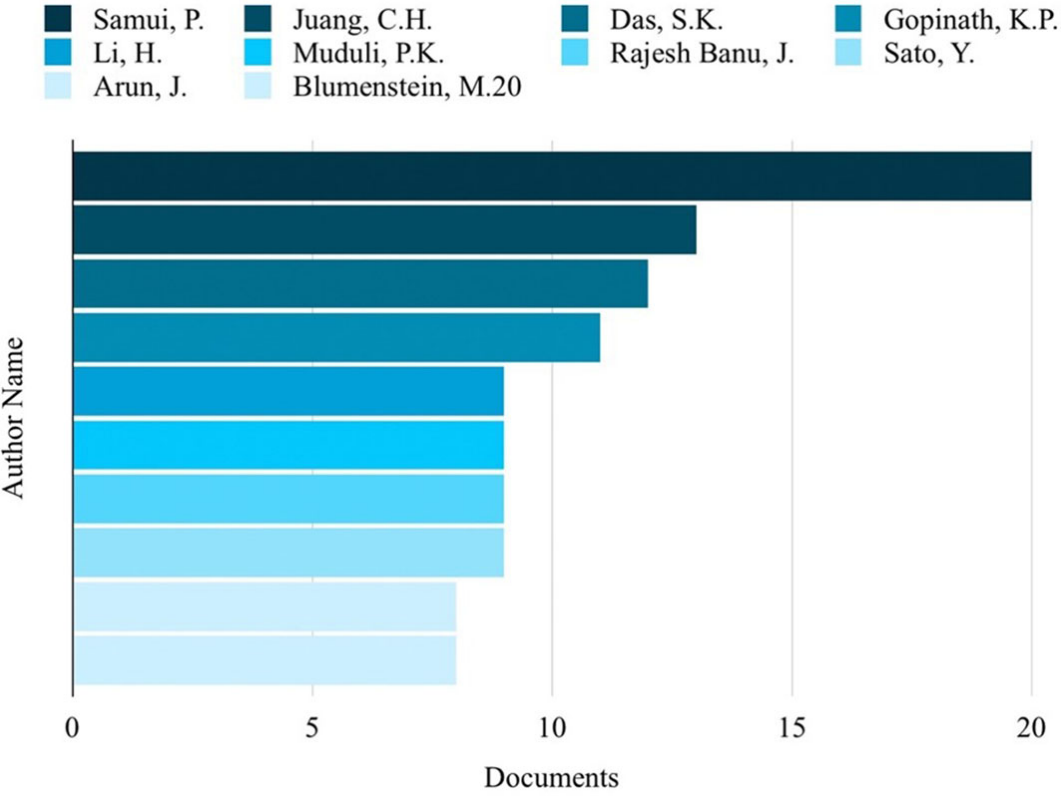
Authors with the most publications related to ML in the HTL process.

**Figure 5.**
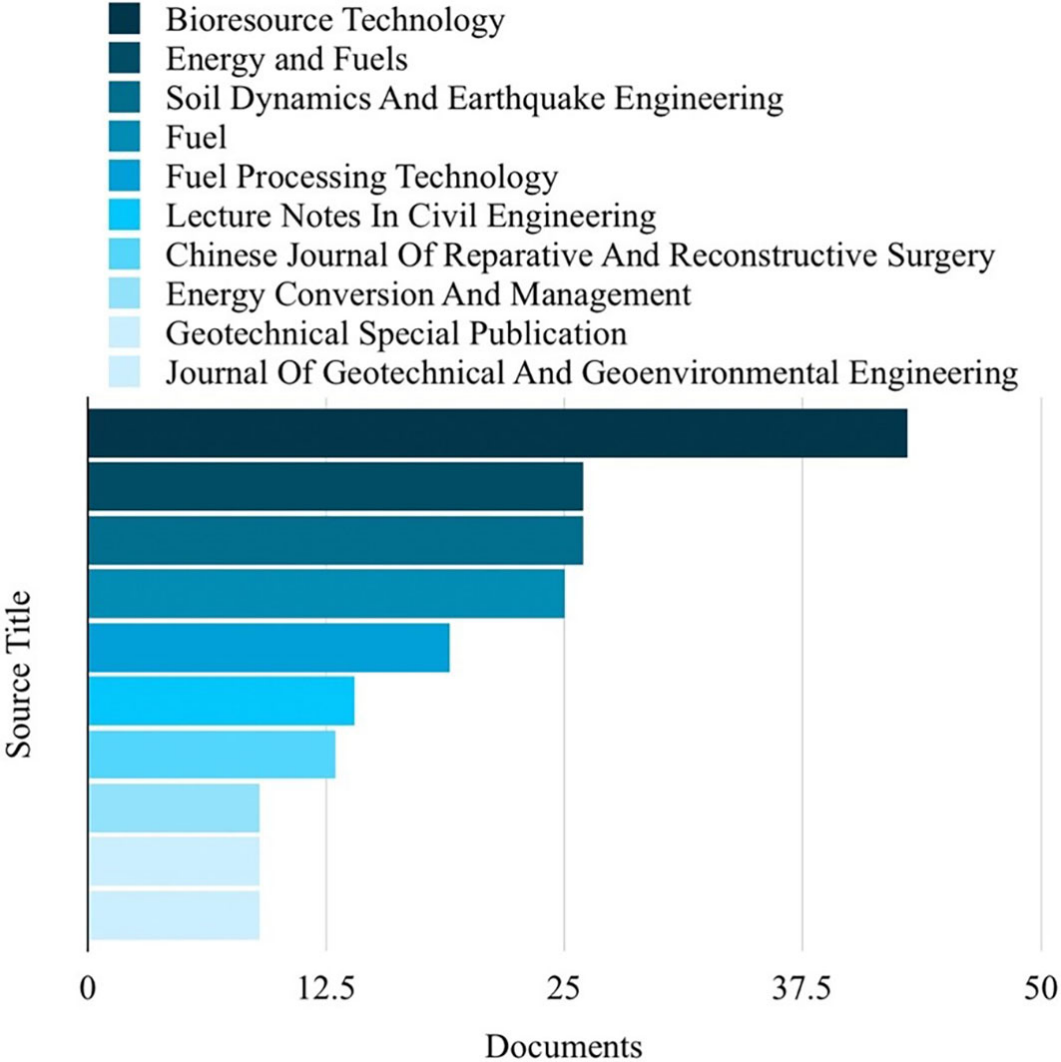
Source titles related to ML in the HTL process.

**Figure 6.**
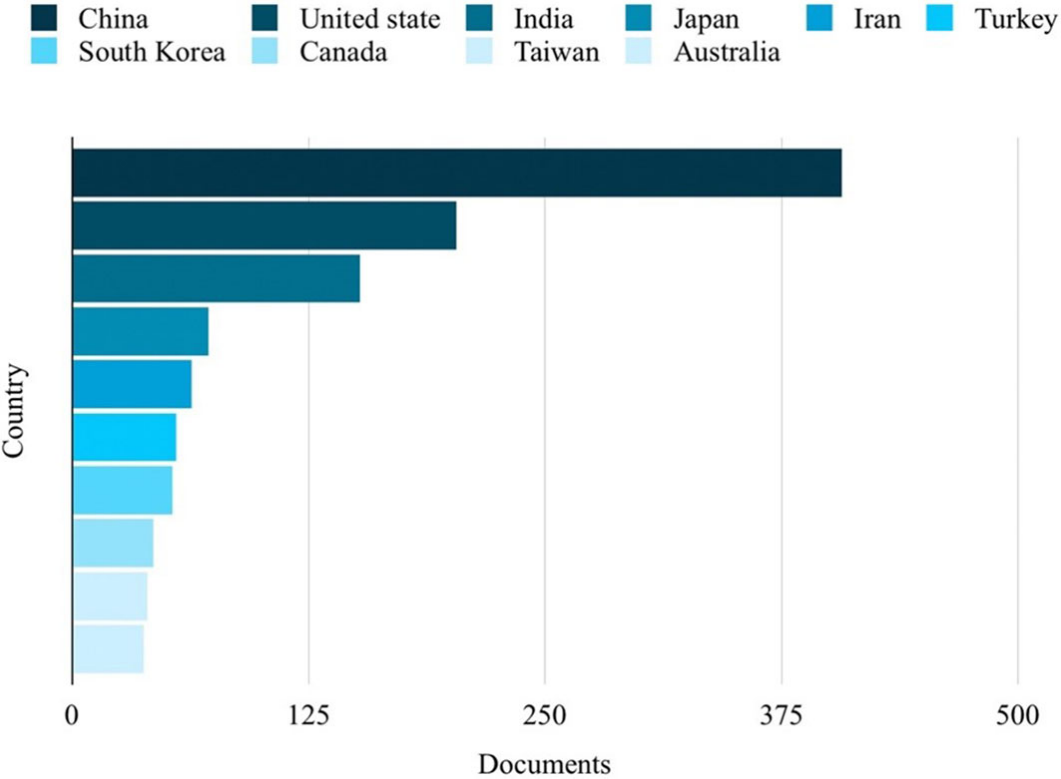
Countries with the most publications related to ML in the HTL process.

Based on the analysis of search results related to ML in the HTL process, it was possible to rank the top 10 in terms of subject area, author name, source title, and country. Engineering emerged as the most published subject area, with 350 publications, as depicted in Figure 3. Among the authors, Samui, P, stood out with the highest number of publications on ML in HTL processes, totaling 20 publications, as shown in Figure 4. The most frequently published source title was “Bioresource Technology,” with a notable 43 publications, as illustrated in Figure 5. China emerged as the country with the highest number of publications on the studied topic, with a total of 407 publications, as depicted in Figure 6.

From Figure 3, it can be seen that China ranks first in publications on ML in HTL processes, which has more than twice that of the second place. Figure 3 reveals a diverse range of disciplines showing interest in studying ML in HTL processes. Engineering emerges as the most engaged subject, constituting 13.5% or 350 publications. Following closely behind are Earth and Planetary Sciences and Energy, accounting for 10.5% or 272 publications. This demonstrates a shift towards adapting to human needs in environmental preservation and exploring clean energy sources through HTL. The application of ML as a tool enables time and resource savings in experimental endeavors, reinforcing its significance in this field. ^22^

### Bibliometric analysis

The bibliometric analysis in this study utilized the VOSviewer program to construct cluster maps and identify relationships within the database. The cluster map construction involved three aspects: co-citation correlation, keyword correlation, and published countries.

**Figure 7.**
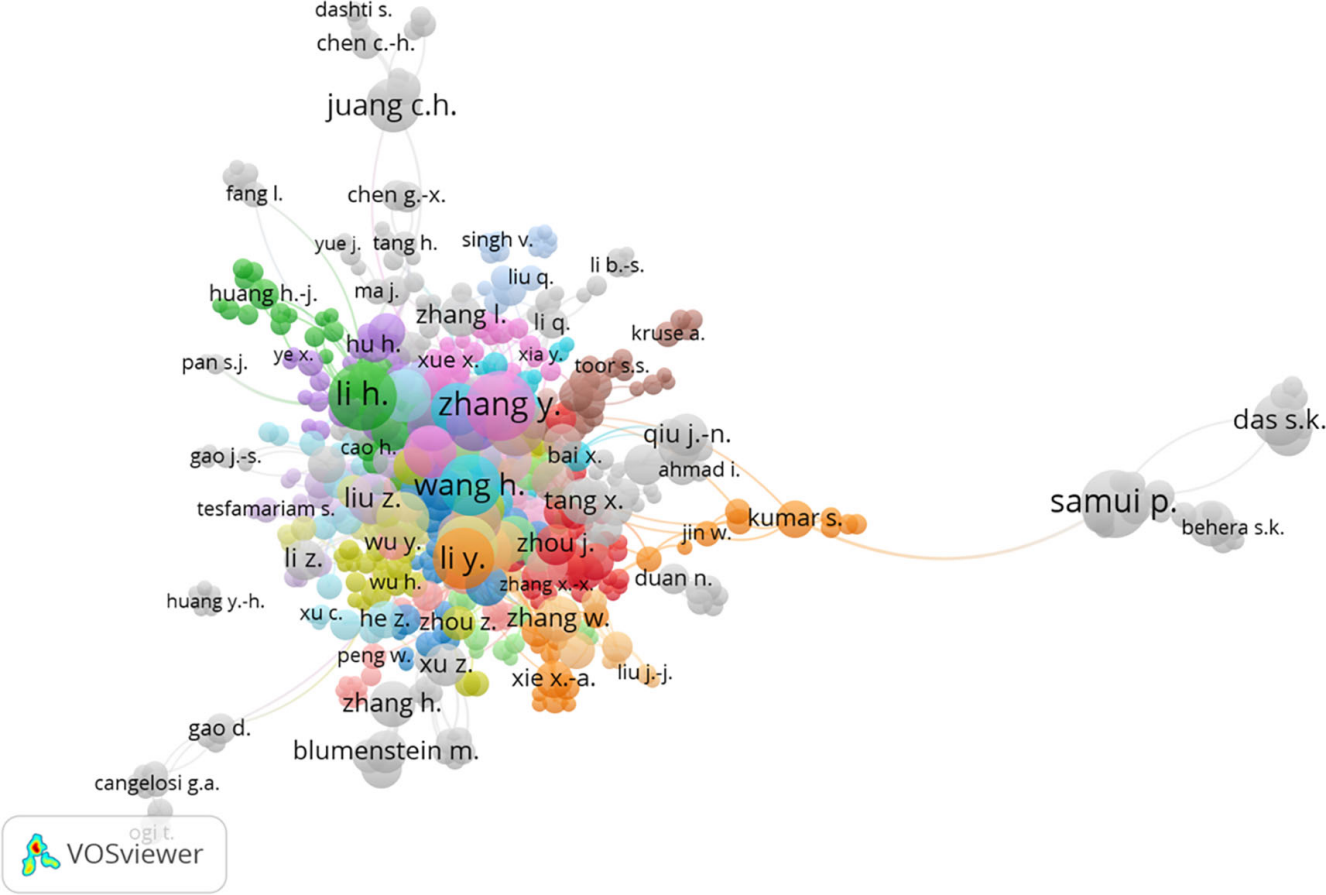
Cluster map based on the co-authorship authors (via Scopus on July 1, 2023).

Figure 7 displays the cluster map based on co-authorship authors, revealing connections among various author groups. A total of 38 author groups were identified. The analysis indicated that Zhang Y. had the highest number of links, with 21 links and 506 citations. However, it was observed that the number of links did not have a significant impact on citations. Li H.I. and Goh A.T.C. had 20 and 6 links, respectively, but garnered 1048 and 877 citations, respectively.

**Figure 8.**
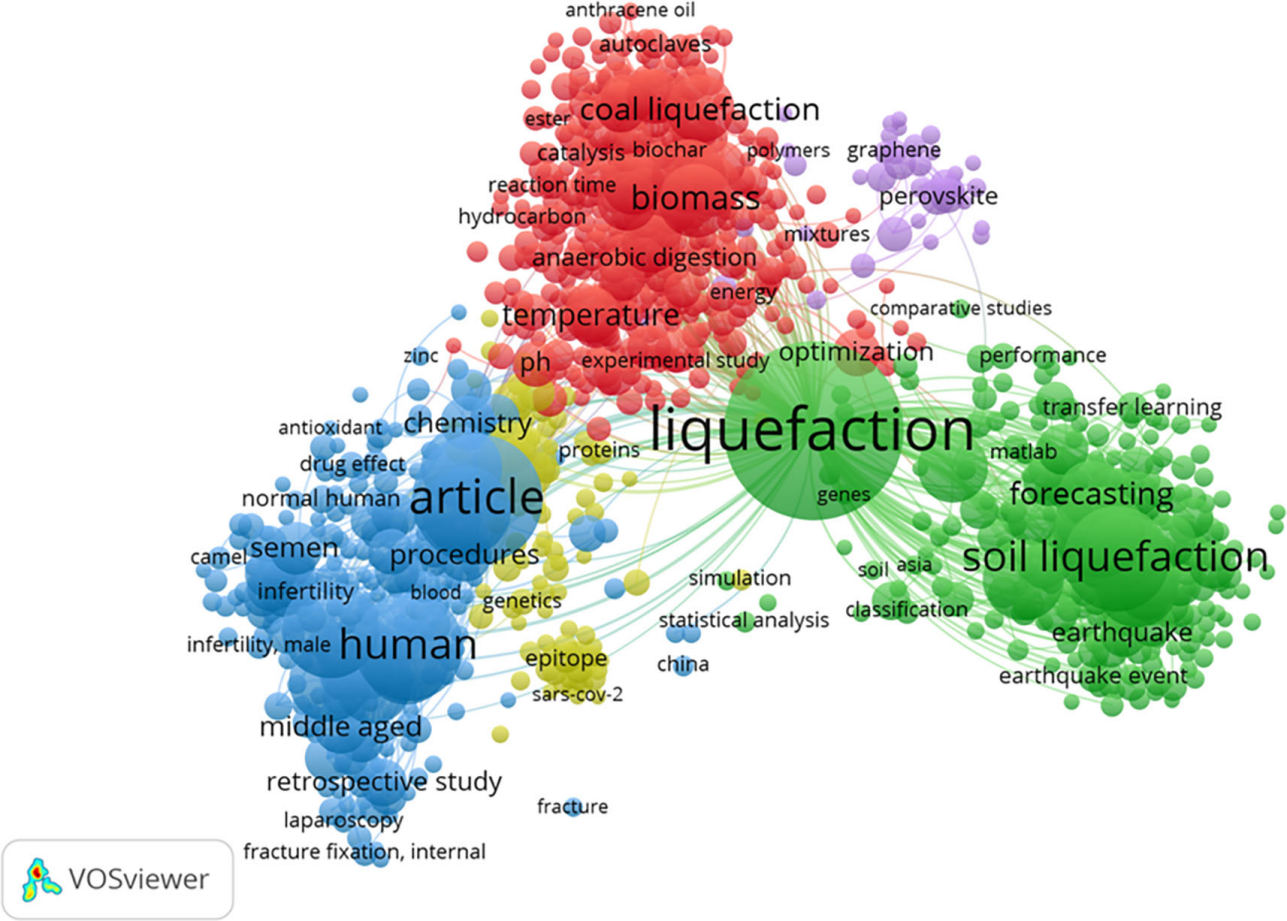
Cluster map based on the co-occurrence all keywords (via Scopus on July 1, 2023).

Figure 8 represents the cluster map based on the co-occurrence of all keywords. It revealed the existence of 5 keyword groups, with a predominant focus on HTL process-related keywords. Interestingly, ML keywords did not form a substantial cluster, potentially due to their relatively lower number compared to the keywords associated with the HTL process chain. The top 5 most common keywords were liquefaction, article, human, soil liquefaction, and male, with frequencies of 590, 308, 219, 203, and 173, respectively. The keyword ML appeared in the 6th position, specifically in the form of “neural networks,” with a frequency of 156.

**Figure 9.**
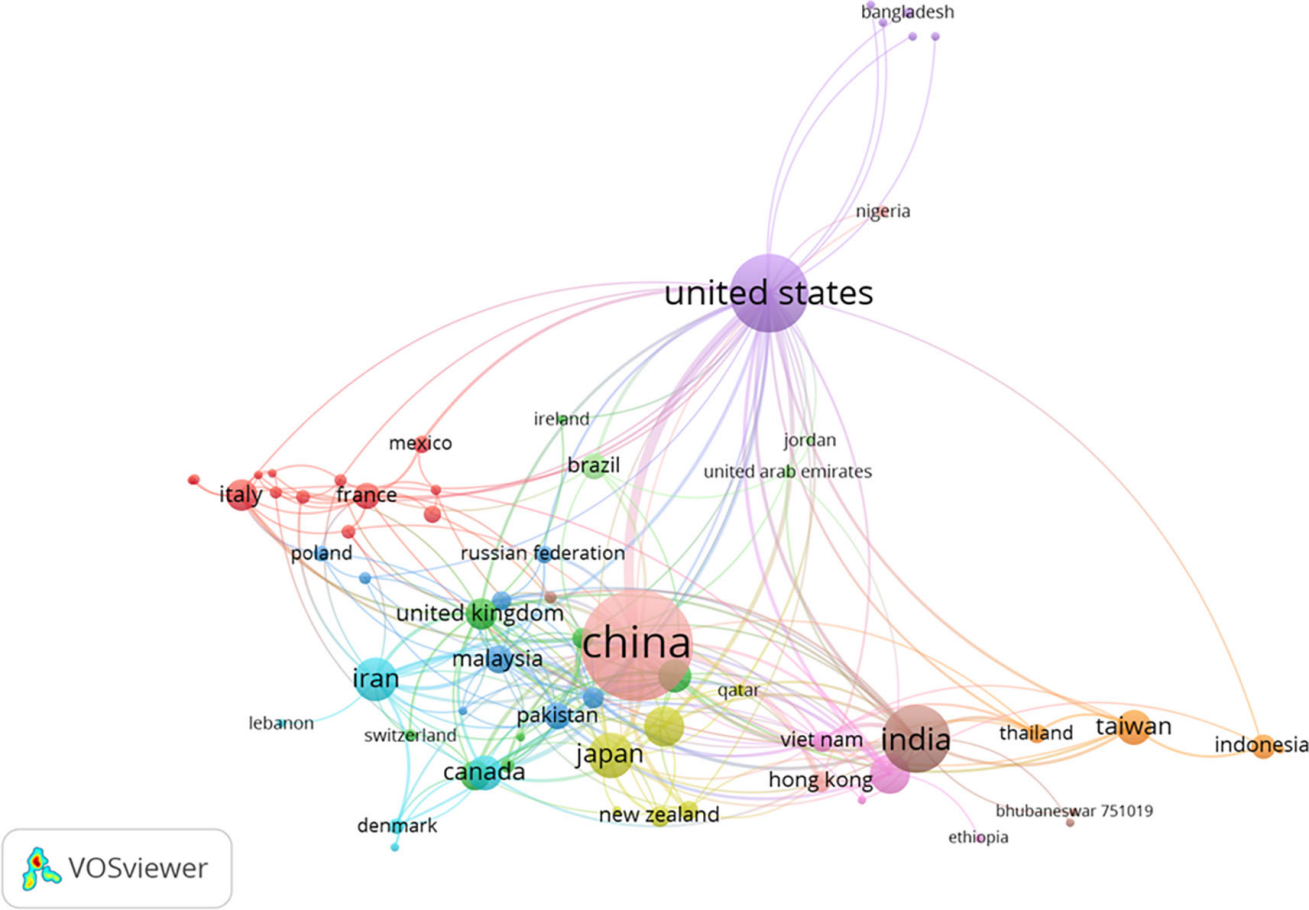
Cluster map based on the co-authorship countries (via Scopus on July 1, 2023).

Figure 9 depicts the cluster map based on co-authorship countries, revealing 13 distinct groupings of countries. Among them, China emerged with the highest number of links, totaling 6,048 citations and 94 links. The United States ranked second in terms of links, accumulating 5,767 citations and 71 links. India secured the third position in links, with 2,193 citations and 39 links.

## Conclusion

The bibliometric analysis conducted in this study revealed a continuous increase in publications related to ML in HTL processes from 1955 to 2023. It was found that China has been the leading country in terms of publishing research in this area. The “Bioresource Technology” journal emerged as the most popular choice for publishing articles on ML in the HTL process. The field of engineering, particularly with a focus on the keyword “Liquefaction,” garnered the most interest among researchers. These findings provide valuable insights and can serve as a guide for future studies on ML in HTL processes, offering a comprehensive overview of the current state of research in this domain.

## Ethical approval

The dataset described in this article does not involve any human subjects, animal experiments, or data collected from social media platforms.

## Author roles

**Katongtung T:** Conceptualization, Data Curation, Formal Analysis, Investigation, Methodology, Project Administration, Validation, Visualization, Writing – Original Draft Preparation; **Sukpancharoen S:** Formal Analysis, Writing – Review & Editing; **Sinthupinyo S:** Formal Analysis, Writing – Review & Editing; **Tippayawong N:** Conceptualization, Supervision, Funding Acquisition, Formal Analysis, Writing – Review & Editing.

## Data Availability

Mendeley Data: Dataset for: Current scenario of machine learning applications to hydrothermal liquefaction via bibliometric analysis, DOI: 10.17632/st4xh92wm2.1. ^35^ Data are available under the terms of the Creative Commons Attribution 4.0 International license (CC-BY 4.0).
