# Supplementary material for: Current scenario of machine learning applications to hydrothermal liquefaction via bibliometric analysis
Source: F1000Res. 2025 Mar 11;13:1131. Originally published 2024 Oct 4. [Version 3] doi: 10.12688/f1000research.156514.3 (PMC12203483; doi:10.12688/f1000research.156514.3)
Supplement: Peer review history

# Reviewer report: Reviewer response for version 3

**DOI:** 10.5256/f1000research.177841.r376870

**Published:** 2025-05-07

**Authors:** Muhammad Raziq Rahimi Kooh

**Affiliations:** 1 Universiti Brunei Darussalam, Darussalam, Brunei

*This work focuses on the bibliographic evaluation of hydrothermal liquefaction using Scopus data. Please see the specific comment below for details.*

1. Minor error (A) ensure all the acronyms are defined on their first appearance. (B) Ensure all the symbols, such as R2 are formatted correctly.
2. Introduction. The section related to ionic liquids does not seem relevant.
3. The table description and figure’s caption should be informative.
4. The lack of discussion is a major concern. While specific countries, journals or authors may have the most HTL publications, what insights can you draw from these? What does all this data mean? Apart from ML, which is a current trend for all domains of science, are there any other specific insights that are obtained from the bibliographic analysis?

Is the work clearly and accurately presented and does it cite the current literature?

Yes

If applicable, is the statistical analysis and its interpretation appropriate?

Not applicable

Are all the source data underlying the results available to ensure full reproducibility?

Yes

Is the study design appropriate and is the work technically sound?

Yes

Are the conclusions drawn adequately supported by the results?

No

Are sufficient details of methods and analysis provided to allow replication by others?

Yes

Reviewer Expertise:

Machine learning

I confirm that I have read this submission and believe that I have an appropriate level of expertise to confirm that it is of an acceptable scientific standard, however I have significant reservations, as outlined above.

# Reviewer report: Reviewer response for version 2

**DOI:** 10.5256/f1000research.176096.r355299

**Published:** 2025-01-09

**Authors:** Lili Qian

**Affiliations:** 1 Jiangsu University, Zhenjiang, Jiangsu, China

This manuscript can be accepted.

Is the work clearly and accurately presented and does it cite the current literature?

Partly

If applicable, is the statistical analysis and its interpretation appropriate?

Partly

Are all the source data underlying the results available to ensure full reproducibility?

Yes

Is the study design appropriate and is the work technically sound?

Partly

Are the conclusions drawn adequately supported by the results?

Yes

Are sufficient details of methods and analysis provided to allow replication by others?

Partly

Reviewer Expertise:

My research focused on hydrothermal liquefaction and machine learning. We submitted a similar paper titled "A Review on Machine Learning-Aided Hydrothermal Liquefaction Based on Bibliometric Analysis" on August this year.

I confirm that I have read this submission and believe that I have an appropriate level of expertise to confirm that it is of an acceptable scientific standard.

# Reviewer report: Reviewer response for version 2

**DOI:** 10.5256/f1000research.176096.r341351

**Published:** 2025-01-03

**Authors:** Venu Babu Borugadda

**Affiliations:** 1 University of Saskatchewan, Saskatoon, Canada

1. Are there a similar papers in the literature with bibliometric analysis ? Please report some with references and explain how these papers are contributing for the scientific/research growth ?

 2. What is the take home message from this bibliometric analysis on HTL and ML?

 3. What are the key challenges of this type of work ?

 4. Besides scopus what are the other potential search engines to make bibliometric analysis ?

 5. Please mention the tables and figures after they are referred to in the text. Many tables and figures are reported before they are being referred.

 6. Fig 3, how ML and HTL are related to the Computer Science, Earth and planetary, Medicine and biochemistry? Please justify or remove them using more filters.

 7. Fig.4, please mention the references of top 10 authors in the reference section.

 8. Page 8 last paragraph, first sentence, it's figure 6, not 3.

 9. Fig.8, why human, soil liquefaction, male keywords are considered ?

Is the work clearly and accurately presented and does it cite the current literature?

Yes

If applicable, is the statistical analysis and its interpretation appropriate?

Not applicable

Are all the source data underlying the results available to ensure full reproducibility?

Partly

Is the study design appropriate and is the work technically sound?

Yes

Are the conclusions drawn adequately supported by the results?

Yes

Are sufficient details of methods and analysis provided to allow replication by others?

Yes

Reviewer Expertise:

Methodology and key question raised in comments 1-5

I confirm that I have read this submission and believe that I have an appropriate level of expertise to confirm that it is of an acceptable scientific standard, however I have significant reservations, as outlined above.

# Response

**Published:** 2025-02-03

**Authors:** Tossapon katongtung

**Affiliations:** Mechanical Engineering, Chiang Mai University, Chiang Mai, Chiang Mai, Thailand

Revised, as suggested.

# Response

**Published:** 2025-02-07

**Authors:** Tossapon katongtung

**Affiliations:** Mechanical Engineering, Chiang Mai University, Chiang Mai, Chiang Mai, Thailand

1.Revised, as suggested. please refer to the Introduction section.Revised, as suggested. please refer to the Introduction section.

 2.As the research on HTL-based ML development is still in its early stages and relatively limited at present, this study fills an important knowledge gap in the field. The outcomes of this analysis will contribute significantly to the formulation of a comprehensive research plan by identifying potential future research directions and fostering collaborative relationships among researchers in this emerging field.

 3.The limitations of this type of research include the validity of the data and the selection of relevant data for presentation.

 4.The Web of Science, Scopus, Dimensions, and PubMed databases can be used to perform bibliometric analyses using VOSviewer.

 5.Revised, as suggested.

 6.This information was obtained from the Scopus database and has not been manipulated, as the dataset was preserved in its original form.

 7.It is not feasible to cite all of the publications in this ranking, as the list includes a substantial number of entries—for example, the top ranking alone comprises 20 publications on the topic. Moreover, citations are unnecessary here because the discussion is based on quantitative variables

 8.Revised, as suggested.

 9.This information was obtained from the Scopus database and has not been manipulated, as the dataset was preserved in its original form.

# Reviewer report: Reviewer response for version 1

**DOI:** 10.5256/f1000research.171837.r341356

**Published:** 2024-12-16

**Authors:** Muntasir Shahabuddin, Andrew Charlebois

**Affiliations:** 1 Chemical Engineering, Worcester Polytechnic Institute, Worcester, MA, USA

**Manuscript Title:** Current scenario of machine learning applications to hydrothermal liquefaction via bibliometric analysis

**Overview:**

 The reviewer would like to thank the editors of F1000 for the opportunity to provide feedback on the manuscript  “Current scenario of machine learning applications to hydrothermal liquefaction via bibliometric analysis”. This study by Katongtung et al. leverages bibliometric analysis to collect and analyze publications relating to machine learning on hydrothermal liquefaction systems in the corpus of existing literature. Using a Scopus search, the authors uncovered the most productive authors in the field, and geopolitical regions with the greatest academic output. While the conclusions of this study (in which Chinese and US scientists tend to be the most productive in this area from a raw studies-published perspective) are useful, the manuscript may benefit from reformatting of figures for better clarity of the data being communicated, and for reconsideration of how each figure contributes to the overall narrative conclusion of the work. In light of the following comments, the reviewer recommends that this work is revised and resubmitted after major revisions, or reconsidered for indexing at another time.

**Major Comments:  **

1. This paper should do a better job to introduce and motivate the problem/question it is trying to solve. The way the information is presented makes the analysis seem very exploratory and not directed. This leads to some of the figures, figure 3 for example, to seem disconnected from the overall story
  1. The work’s motivation reads well and is well cited. I would recommend quantifying the conditions HTL occurs at (i.e. the exact pressures and temperature ranges rather than qualitative descriptions of “medium” and “high” temperature and pressure respectively. I would also recommend discussing why the higher heating values are useful – the authors discuss “various applications” but may consider discussing the exact applications (for example in aviation fuels) which may bolster the narrative.
  2. In the third paragraph, in which an ML model and AI as a whole are referred to vaguely as “the learning component of a machine” or in comparison to a “brain” may risk unsubstantiated analogies – rather than exhibiting “intelligent behavior” many applications of machine learning in HTL are rather used as fitting tools that can predict metrics that are empirically useful. I would recommend discussing ML and AI in a more objective way. Overall, the description of ML and AI in the third introduction paragraph risk miscommunicating how ML and AI models work and often self contradict – for example, the authors refer to ML as not programs, but in the same sentence, refer to humans programming the ML. This kind of confusion warrants a more mechanistic description of ML and AI.
  3. The reviewer appreciates the well cited and discussed summary of ML use in the introduction. However, many of the cited works span a broad range of use cases – for example, HTL pretreatments, yield prediction, and specific feedstock compositions are discussed, but the reviewer worries that this may be casting too wide of a net to focus on the nature of this study. The authors may stylistically disagree as needed.
2. Keyword choice may benefit from some honing – the terms may be too broad to reach the correct audience for what is a relatively niche intersectional field. The reviewer recommends specifying some of the keywords – even including HTL seems like great choice.
3. Overall, while the authors are able to clearly communicate how the work was performed, the results and the specific conclusions to be garnered from each figure could be better communicated. The reviewer struggles to find specific conclusions or a line of narrative information that the figures tie into. This poises the paper as primarily exploratory rather than providing the incisive synthesis of information that may be interpreted from the data in the figures. I highly recommend visualizing some of this useful data in a manner that a reader can derive obvious conclusions the authors wish to communicate (for example for the graph/node plot, greater spacing between the nodes may aid clarity). Similarly, I would recommend poising a stronger line of thought tying each point (i.e. each figure) together.

**Other Comments: **R ^2^ vs MAE, RMSE, error metrics

1. In paragraph 4 of the introduction, R ^2^ is used to refer to both accuracy and precision when referring to machine learning models. While R ^2^ is an empirically useful metric to quantify the correlation between two variables or goodness of fit, it does not describe accuracy or precision. When talking about ML models, metrics that communicate the applied usefulness or “error bounds” of a model may be more useful, and are widely used throughout ML in HTL. Some examples include RMSE (root mean squared error) or MAE (mean absolute error). R ^2^ can be used to describe the performance of the model (how close to a straight line does the parody plot of the model create)
2. Regarding figure clarity, the reviewer suggests using colors of higher contrast – they are currently set up to be in the form of a heat map, where they are similar to each other but change down the list. This implies a relationship between the different items on the figures that may not exist (and if it does it could be helpful to clarify it!) Use of more distinct colors may rectify this

Is the work clearly and accurately presented and does it cite the current literature?

Partly

If applicable, is the statistical analysis and its interpretation appropriate?

Not applicable

Are all the source data underlying the results available to ensure full reproducibility?

Yes

Is the study design appropriate and is the work technically sound?

No

Are the conclusions drawn adequately supported by the results?

Yes

Are sufficient details of methods and analysis provided to allow replication by others?

Yes

Reviewer Expertise:

Techno-economics, hydrothermal liquefaction, machine learning, electrochemical engineering, reactor engineering

We confirm that we have read this submission and believe that we have an appropriate level of expertise to state that we do not consider it to be of an acceptable scientific standard, for reasons outlined above.

# Response

**Published:** 2024-12-16

**Authors:** Tossapon katongtung

**Affiliations:** Mechanical Engineering, Chiang Mai University, Chiang Mai, Chiang Mai, Thailand

Thank you for your advice, I will revise the manuscript as per the instructions and resubmit it through the system.

# Response

**Published:** 2024-12-17

**Authors:** Tossapon katongtung

**Affiliations:** Mechanical Engineering, Chiang Mai University, Chiang Mai, Chiang Mai, Thailand

1. This paper should do a better job to introduce and motivate the problem/question it is trying to solve. The way the information is presented makes the analysis seem very exploratory and not directed. This leads to some of the figures, figure 3 for example, to seem disconnected from the overall story

 = Thank you for the suggestion; however, we believe Figure 3 remains important as it highlights the most relevant study on this subject and helps readers identify gaps in the research within this field.

 2.The work’s motivation reads well and is well cited. I would recommend quantifying the conditions HTL occurs at (i.e. the exact pressures and temperature ranges rather than qualitative descriptions of “medium” and “high” temperature and pressure respectively. I would also recommend discussing why the higher heating values are useful – the authors discuss “various applications” but may consider discussing the exact applications (for example in aviation fuels) which may bolster the narrative.

 = Revised, as suggested. please refer to lines 57-63 in Introduction section.

 3. In the third paragraph, in which an ML model and AI as a whole are referred to vaguely as “the learning component of a machine” or in comparison to a “brain” may risk unsubstantiated analogies – rather than exhibiting “intelligent behavior” many applications of machine learning in HTL are rather used as fitting tools that can predict metrics that are empirically useful. I would recommend discussing ML and AI in a more objective way. Overall, the description of ML and AI in the third introduction paragraph risk miscommunicating how ML and AI models work and often self contradict – for example, the authors refer to ML as not programs, but in the same sentence, refer to humans programming the ML. This kind of confusion warrants a more mechanistic description of ML and AI.

 = Revised, as suggested. The text that may have caused confusion for readers has been removed.

 4. The reviewer appreciates the well cited and discussed summary of ML use in the introduction. However, many of the cited works span a broad range of use cases – for example, HTL pretreatments, yield prediction, and specific feedstock compositions are discussed, but the reviewer worries that this may be casting too wide of a net to focus on the nature of this study. The authors may stylistically disagree as needed.

 = Based on the explanation provided in the introduction, the authors believe that presenting examples of research in various forms and across a wide range will help readers gain a clearer understanding of the overall scope of ML. Therefore, the author suggests retaining all the original sentences in the manuscript.

 5.Keyword choice may benefit from some honing – the terms may be too broad to reach the correct audience for what is a relatively niche intersectional field. The reviewer recommends specifying some of the keywords – even including HTL seems like great choice.

 = Revised, as suggested. please refer to the red text in the Keywords section.

 6. Overall, while the authors are able to clearly communicate how the work was performed, the results and the specific conclusions to be garnered from each figure could be better communicated. The reviewer struggles to find specific conclusions or a line of narrative information that the figures tie into. This poises the paper as primarily exploratory rather than providing the incisive synthesis of information that may be interpreted from the data in the figures. I highly recommend visualizing some of this useful data in a manner that a reader can derive obvious conclusions the authors wish to communicate (for example for the graph/node plot, greater spacing between the nodes may aid clarity). Similarly, I would recommend poising a stronger line of thought tying each point (i.e. each figure) together.

 = The bibliometric analysis in this study aims to provide an overview and identify trends in related research, serving as a guide for readers pursuing further studies on this topic. The authors believe that the statistical data and related information have been presented comprehensively.

 7. In paragraph 4 of the introduction, R ^2^is used to refer to both accuracy and precision when referring to machine learning models. While R ^2^ is an empirically useful metric to quantify the correlation between two variables or goodness of fit, it does not describe accuracy or precision. When talking about ML models, metrics that communicate the applied usefulness or “error bounds” of a model may be more useful, and are widely used throughout ML in HTL. Some examples include RMSE (root mean squared error) or MAE (mean absolute error). R ^2^ can be used to describe the performance of the model (how close to a straight line does the parody plot of the model create)

 = Revised, as suggested. please refer to the RED text in lines 88-89, 96 and 113-114. 

 8. Regarding figure clarity, the reviewer suggests using colors of higher contrast – they are currently set up to be in the form of a heat map, where they are similar to each other but change down the list. This implies a relationship between the different items on the figures that may not exist (and if it does it could be helpful to clarify it!) Use of more distinct colors may rectify this

 = Thank you for the advice. However, the authors intended for the images in the work to follow the same color theme. Additionally, the colors in the original image were chosen to represent intensity, where stronger colors indicate greater values and lighter colors indicate lower values, similar to contouring.

# Reviewer report: Reviewer response for version 1

**DOI:** 10.5256/f1000research.171837.r330069

**Published:** 2024-10-24

**Authors:** Lili Qian

**Affiliations:** 1 Jiangsu University, Zhenjiang, Jiangsu, China

This manuscript performed bibliometric analysis of machine learning applications to hydrothermal liquefaction. However, similar studies have been reported and the following issues should be addressed. A major revision is needed before the indexing.

 1 Background in abstract: It is important to emphasize HTL's focus on wet biomass.

 2 Methods in abstract: machine learning should be introduced.

 3 Second paragraph in introduction: Machine learning has emerged as the most popular technology to address such difficult and complex problems efficiently. What specifically do “such difficult and complex problems” refer to?

 4 Fourth paragraph in introduction: There is too much information about ionic liquids.

 5 Fifth paragraph in introduction: The application of the bibliometric method in the HTL field has not been introduced, nor has its application in the ML field.

 6 “Based on the available literature review, this study represents the first bibliographic analysis conducted to evaluate research trends specifically in the development of ML within the context of HTL.”. It is not the first bibliographic study on ML-based HTL. similar studies have been reported. What is the novelty of the manuscript?

 7 Methods: The first paragraph in methods is the introduction of bibliometrics. Move it to the introduction part.

 8 Figure 8: why “article” is a keyword?

 9 No reference in this year.

Is the work clearly and accurately presented and does it cite the current literature?

Partly

If applicable, is the statistical analysis and its interpretation appropriate?

Partly

Are all the source data underlying the results available to ensure full reproducibility?

Yes

Is the study design appropriate and is the work technically sound?

Partly

Are the conclusions drawn adequately supported by the results?

Yes

Are sufficient details of methods and analysis provided to allow replication by others?

Partly

Reviewer Expertise:

My research focused on hydrothermal liquefaction and machine learning. We submitted a similar paper titled "A Review on Machine Learning-Aided Hydrothermal Liquefaction Based on Bibliometric Analysis" on August this year.

I confirm that I have read this submission and believe that I have an appropriate level of expertise to confirm that it is of an acceptable scientific standard, however I have significant reservations, as outlined above.

# Response

**Published:** 2024-10-24

**Authors:** Tossapon katongtung

**Affiliations:** Mechanical Engineering, Chiang Mai University, Chiang Mai, Chiang Mai, Thailand

**Thank you for your advice. I can answer the following questions you raised.**

 1.Background in abstract: It is important to emphasize HTL's focus on wet biomass.

**Response: **Revised, as suggested.

 2.Methods in abstract: machine learning should be introduced.

**Response: **Revised, as suggested.

 3 Second paragraph in introduction: Machine learning has emerged as the most popular technology to address such difficult and complex problems efficiently. What specifically do “such difficult and complex problems” refer to?

**Response: **This sentence refers to applying ML to processes that are difficult to explain, in this case HTL processes, as in the paragraph, and I believe the following sentence answers this question. “It offers promising avenues for understanding and optimizing HTL processes through data-driven analysis and predictive modeling.” If the reader wishes to elaborate on this, I have added some sentences to the manuscript.

 4 Fourth paragraph in introduction: There is too much information about ionic liquids.

**Response: **Revised, as suggested.

 5 Fifth paragraph in introduction: The application of the bibliometric method in the HTL field has not been introduced, nor has its application in the ML field.

**Response: **In this sentence, I am confident that I wrote it correctly at that time, because I had been studying this issue since June 2023 and had tried to submit it to a journal for publication but did not receive a response, so I withdrew it and submitted it to F1000research in September 2024. I have kept all my submissions from June 2023. If you want to see them, I can send them to you.

 6 “Based on the available literature review, this study represents the first bibliographic analysis conducted to evaluate research trends specifically in the development of ML within the context of HTL.”. It is not the first bibliographic study on ML-based HTL. similar studies have been reported. What is the novelty of the manuscript?

**Response: **I think we have similar ideas on how to work on this, but I must say that at the time I wrote this study, there was no research on this subject before.

 In this sentence, I am confident that I wrote it correctly at that time, because I had been studying this issue since June 2023 and had tried to submit it to a journal for publication but did not receive a response, so I withdrew it and submitted it to F1000research in September 2024. I have kept all my submissions from June 2023. If you want to see them, I can send them to you. I have submitted Case Studies in Chemical and Environmental Engineering Manuscript Number: CSCEE-D-23-00310 on June 30, 2023.

 7 Methods: The first paragraph in methods is the introduction of bibliometrics. Move it to the introduction part.

**Response: **I think this sentence should remain in its original place and should not be changed.

 8 Figure 8: why “article” is a keyword?

**Response: **I collected data from Scopus database and did not add any data to the dataset.

 9 No reference in this year.

**Response: **I did this study before 2024 and I gave a clear timeline in the manuscript.
